# Design of a prospective study on mental health and quality of life of maltreated children (aged 5–16 years) after a report to an advice and reporting center on child abuse and neglect

**DOI:** 10.1186/1471-2458-13-942

**Published:** 2013-10-09

**Authors:** Froukje Snoeren, Cees Hoefnagels, Silvia MAA Evers, Francien Lamers-Winkelman

**Affiliations:** 1 Netherlands Institute of Mental Health and Addiction, Trimbos Institute, PO Box 725, Utrecht, 3500 AS, Netherlands; 2 CAPHRI, School for Public Health and Primary Care, Maastricht, Netherlands; 3 VU University, Amsterdam, Netherlands

**Keywords:** Child maltreatment, Mental health, Quality of life, Prospective design, Advice and reporting agency on child abuse and neglect

## Abstract

**Background:**

Child maltreatment is recognized as a widespread problem with huge implications for mental health and quality of life. Studies have repeatedly shown that victims of child maltreatment report significantly more adverse life outcomes than non-victims. The main objective of the study is (1) to examine the mental health and quality of life of maltreated children over a 1.5 year period beginning shortly after a report has been filed with an Advies- en Meldpunt Kindermishandeling (AMK) (advice and reporting center on child abuse and neglect). Secondary objectives are: (2) to examine how relevant determinants influence the mental health and quality of life of maltreated children, and (3) to examine differences in mental health and quality of life outcomes when comparing families of Dutch origin with families originating from Morocco and Suriname.

**Methods/Design:**

A prospective study will be performed, in which parent–child dyads will be followed over a 1.5 year period. Participants will be recruited shortly after the report to the AMK and they will be asked to complete a questionnaire four times, at baseline and every six months thereafter. Data will be analyzed using a longitudinal multi-level analysis.

**Discussion:**

The study is expected to yield evidence about the mental health and quality of life of maltreated children and about determinants that influence their mental health and quality of life outcomes. Strengths of this study are (1) the design which makes it possible to start examining outcomes shortly after or even during the actual maltreatment and to follow parent–child dyads for 1.5 years, and (2) asking children as informants about their own situation by making use of self-report questionnaires as much as possible. Limitations include the risks of selection bias and loss to follow-up during 1.5 years of data collection.

**Trial registration:**

NTR3674, funded by ZonMw, project 15700.2012.

## Background

There is increasing evidence to support the association between child maltreatment and adverse life outcomes during childhood as well as adulthood [1,2]. It is generally known that victims of maltreatment report significantly more short- and long-term mental health problems than non-victims [3-7]. In the last few decades, quality of life has become an important topic of research in the field of childhood maltreatment. Studies have shown that adults who have been maltreated as children report lower quality of life than adults without an abusive past [8-10]. The concept of quality of life adds a subjective, self-reported component to objective, clinical measures and has been successfully used in mental health related research [11-13] and in clinical practice [14]. However, there has not been much quality of life research focusing on children [15,16], especially maltreated children [17]. It is therefore important to study the quality of life of maltreated children and the determinants that influence their quality of life [15,16].

A recent Dutch study monitoring the prevalence of all types of child maltreatment in the Netherlands estimated that the number of children and adolescents aged between 0 and 18 years who are exposed to child abuse and neglect was 118,000 (34/1,000 children) in 2010 [18]. Like most Western countries, the Netherlands has agencies where suspected child maltreatment can be reported by professionals, such as teachers and general practitioners, as well as non-professionals such as relatives and neighbors. In recent decades, the annual number of reports of suspected child maltreatment to these AMKs has increased, now numbering over 19,000, which is 16% of the estimated annual exposure to child maltreatment [18]. The AMKs investigate each report of suspected child maltreatment and if the report is substantiated, the AMK refers the child and the family to voluntary child welfare and/or mental health organizations. If voluntary care is rejected, the AMK can report the family to child protection services that can take court action or arrange the child to be placed in care [19]. In view of the finding that longer duration of child maltreatment is associated with poorer outcomes, it is expected that if reporting leads to maltreatment being ended, or sufficient care being provided for child and family, this will benefit children’s mental health and quality of life, or at least contribute to minimizing adverse outcomes [4,20-23]. Although the AMKs play an important role in the Netherlands, the potential benefits and harms of AMK involvement on children’s mental health and quality of life has not yet been studied.

The Dutch society includes large numbers of people with non-Dutch ethnic backgrounds and a substantial part (35% in 2012) of the maltreatment reports concern families of non-Dutch origin. Miller and Cross [24] found differences in the perception of child maltreatment by different ethnic groups, which underlines the importance of examining differences in mental health and quality of life outcomes between children from families of Dutch origin and children from families of non-Dutch origins.

The main objective of the proposed study is to examine the mental health and quality of life of maltreated children over a 1.5-year period beginning shortly after the maltreatment has been reported to an AMK. Many studies on child maltreatment have examined (health) outcomes several years after the maltreatment occurred. This may lead to underreporting of childhood adversities due to memory problems, for example dissociation [10,25]. There is a lack of studies examining outcomes shortly after or during the actual abuse, even though short-term outcomes may be expected to differ from outcomes years later.

Secondly, this study will explore how several relevant determinants influence children’s mental health and quality of life outcomes. These determinants include children’s perceived stress and social support, coping and self-esteem. Research findings have shown discrepancies between child and proxy (i.e. parent) reports when measuring subjective determinants [26-29]. Parents might underreport problems due to lack of knowledge about children’s health, and parental perspectives might be influenced by their own experiences or perceptions [27,30,31]. As a consequence, the value of obtaining children’s self-reports is increasingly recognized, and this study will therefore use child self-reports as a major outcome measurement. In addition, determinants for parents, such as perceived social support and stress, gender-related stress, coping, life events, subjective and objective neighborhood characteristics and alcohol consumption will be examined [32,33].

Thirdly, this study will examine differences in mental health and quality of life outcomes between children from families of Dutch origin and children from families originating from Morocco and Suriname. This study aims to categorize families of non-Dutch origin by ethnicity. The two most prevalent groups of non-Dutch people reported to AMKs are families with a Moroccan background and families with origins in Suriname, which featured in 4.9% and 4.1%, respectively, of all AMK reports in 2012 (Personal communication, A. Verburg, Jeugdzorg Nederland, July 4, 2013).

## Methods/Design

### Objective

The main objective of the study is (1) to examine the mental health and quality of life of maltreated children over a 1.5 year period beginning shortly after the maltreatment has been reported to an AMK. Secondary objectives are: (2) to examine how theoretically and practically relevant determinants influence the mental health and quality of life of maltreated children, and (3) to examine differences in mental health and quality of life outcomes between families of Dutch origin and families originating from Morocco and Suriname.

### Study design

**Figure 1:**
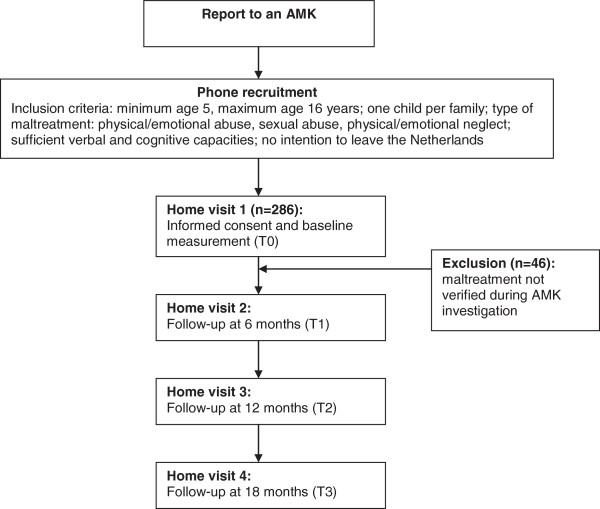
Flow chart of study design.

To answer the research questions, a prospective study will be performed in which parent–child dyads will be asked to complete a questionnaire on four occasions over a 1.5 year period. Baseline assessment will take place shortly after the child maltreatment report is filed with an AMK. Parent–child dyads will be approached for follow-up assessments every six months. Figure 1 presents the flow chart of the study.

### Assessment

Parent–child dyads will be asked to complete a questionnaire four times. Baseline assessment will take place within three months after the report to the AMK. The follow-up assessments will take place every six months. A member of the research team will be present to provide assistance. If necessary, a Moroccan translator can be present as well. Since the official language in Suriname is Dutch, no translator is needed for these participants. The method used to complete the questionnaire will be discussed with the parent and child, and will depend on the participants’ reading and writing skills. If reading and writing skills are poor, the research team member will read the questions out loud and write down the participant’s answer on the questionnaire form. If reading and writing skills are sufficient, participants may choose to complete the questionnaire by themselves. To prevent the parents from influencing the children’s answers, parents and children will be requested to complete their questionnaires in separate rooms. Respondents will be rewarded for their participation by a 10 euro gift voucher to the parents, while the children will receive either a 5 euro gift voucher (children > 10) or an age-appropriate present (5–10 years).

### Target population

Children aged between 5 and 16 years and their primary caretaker will be recruited to participate in this study shortly after the report is filed with the AMK. Children younger than 5 years old are excluded from the study, as this study will rely on self-report as much as possible. Studies have shown that children from the age of 5 are able to reliably self-report on their own health status [34]. Only one child per family will be asked to participate. When a report to the AMK relates to more than one child of the same family, the oldest child within the age range will be included. Additional criteria for inclusion in the study are: (1) a report to an AMK about physical and/or emotional abuse, physical and/or emotional neglect and/or sexual abuse, (2) sufficient verbal and cognitive capacities of both parent and child, as the study will use mainly self-report methods for data collection, and (3) no intention to leave the Netherlands within the next 1.5 years, in view of the follow-up period. All families meeting these criteria will be approached by phone within three months after the report to the AMK. During this phone conversation, an AMK employee will screen the potential participants for verbal and cognitive capacities.

This study will focus only on children for whom maltreatment is verified during the AMK investigation. The maltreatment verification status will be established in a multi-disciplinary meeting at the end of the AMK investigation. At the time when eligible participants are first approached by phone, most reports will still be under AMK investigation, so the families’ maltreatment status will not be known at the time of study inclusion. Parent–child dyads for whom maltreatment is not confirmed, or dyads that are given a 'no maltreatment’ status, will be excluded from the study sample before the first follow-up assessment six months later.

### Setting; dutch advice and reporting centers on child abuse and neglect

Data will be collected from families who have been reported to an AMK. There are seventeen AMKs in the Netherlands, which can be contacted either for professional advice or to report suspected child maltreatment. This study will focus only on reports to the AMKs. The Netherlands has a voluntary reporting system. Reports are discussed in a multidisciplinary team which draws up a plan for investigation to discover if the report is substantiated. In this investigation, the AMK collects information from adults (e.g. parents, teachers, general practitioners) and children (aged 6 years and older) to discover the existence of family problems, the need for child and family care and the willingness to accept care. Based on this information, the multidisciplinary team evaluates the family situation and develops an intervention plan to end maltreatment and to refer to voluntary care. If voluntary care is rejected, the AMK can report the family to child protection services that can take court action or arrange for the child to be placed in care. The AMK has a maximum of 13 weeks to investigate a report of suspected child maltreatment [19].

### Recruitment and sample size

Parent–child dyads will be recruited from seven AMKs in the Netherlands. All AMKs in the four largest cities (Amsterdam, Rotterdam, The Hague, and Utrecht) will be approached to participate in the recruitment for the study, in view of their high percentage of residents of Moroccan and Suriname origin. An additional three large AMKs will be asked to take part in the recruitment phase of the study, taking the level of urbanization into account, to ensure that the sample is representative of Dutch society. The study will focus on children aged between 5 and 16 years, as approximately 70% of all reports to AMKs concern children in this age range [35]. In addition, the study will focus on reports to AMKs of physical and/or emotional abuse, physical and/or emotional neglect and/or sexual abuse, which account for about 70% of all reports to AMKs [35]. Families that meet the inclusion criteria will be approached by phone by an AMK employee within three months after the report was filed with the AMK. In this phone call, they will provide general information about the study, and will invite one parent and one child per family to participate.

Based on expert opinion, we expect a participation rate of 5%. This low expected rate is due to the delicate subject of the study: participants will be recruited shortly after a suspected child maltreatment report, so people might refuse to participate due to the perceived accusation and/or perceived stigmatization and/or stress because of the report to the AMK. In addition, we are expecting obstacles in the recruitment process, for instance because there is often no phone number in the AMK records, people may not be available when the phone call is made, people may not be able to speak Dutch. If a parent–child dyad is willing to participate, an appointment with a member of the research team will be made at a time and place of the families’ choosing (which in most cases will probably be a home visit). During this visit, prior to starting to complete the questionnaire, parents and children will be asked to give written informed consent for their participation. Parents will also be required to give informed consent for the participation of their child.

After the baseline assessment, an estimated 40% of the participants are expected to be lost to follow-up. To minimize loss to follow–up, parents and children will be asked to write down various types of contact information on the informed consent form, e.g. both home and mobile phone numbers, and email addresses. If phone numbers are no longer valid at follow-up, an email or letter will be sent to the last known home address with the request to contact the research team. In case of no response, a member of the research team will visit the family at their last known address to check if they have moved.

This study aims to examine differences in outcomes when comparing children from families of Dutch origin with children from families originating from Morocco and Suriname. To prevent numbers that are too small to allow a useful analysis, we will oversample families originating from Morocco and Suriname. To this end, extra time will be invested in reaching these families, even beyond the times of the day when AMK employees would normally make recruitment calls. A separate list with phone numbers of eligible Moroccan and Suriname families will be printed, and a Moroccan AMK employee will call the Moroccan families to minimize the risk of refusal to participate due to a possible language barrier. Overall, we expect about 150 parent–child dyads to be available for the last assessment 1.5 years after the report to the AMK.

### Primary outcomes measures

#### *Mental health*

The children’s mental health (in terms of internalizing and externalizing psychological problems) will be measured with the Dutch version of the Child Behavior Checklist (CBCL) [36]. This questionnaire will be completed by the parent. Parents will be asked to what extent they observe various behavioral and emotional problems in their child. The CBCL uses a 3-point scale and consists of 113 items. Internal consistency is good (Table 1).

**Table 1:** Internal consistency of the Dutch translations of the questionnaires used to measure child determinants

**Outcomes measure**	**Age group**	**Questionnaire**	**Cronbach’s alpha**	**Reference**
Mental health	Parent proxy	CBCL	.97	Verhulst, [36]
Quality of life	12–16	PedsQL	.85	Engelen et al. [34]
	8–11	PedsQL	.82	Engelen et al. [34]
	5–7	PedsQL	.85	Engelen et al. [34]
Perceived social support	12–16	SSL youngster version	.78	Hoefnagels et al., [38]
	8–11	PSSQ8-11	.73	Snoeren & Hoefnagels, [39]
	5–7	PSSQ subscale negative interactions	.57	Internal report (unpublished)
Perceived stress	12–16	MUSIC	.85	Hoefnagels et al. [38].
	8–11	PSQ8-11	.85	Snoeren & Hoefnagels, [39]
	5–7	PSQ5-7	.60	Internal report (unpublished)
Coping	12–16	CISS	.82 - .87	Evers et al. [60]
	8–11	CSLK	.72 - .88	Boo & Wicherts. [61]
Self-esteem	12–16	CBSA	.66 - .88	Treffers et al. [45]
	8–11	CBSK	.74	Veerman et al. [46]

#### *Quality of life*

Quality of life will be measured with one of three age-appropriate versions (5–7, 8–11, 12–18 years) of the Dutch translation of the Pediatric Quality of Life Inventory (PedsQL) [34]. Children will be asked to express their concerns on the dimensions of physical health and psychosocial health, the latter consisting of the sub dimensions of emotional functioning, social functioning and school functioning. The overall quality of life score will be obtained by adding up the scores on all dimensions. The PedsQL uses a 5-point scale (or a 3-point scale for the 5–7 version) and consists of 23 items. Internal consistency is good (Table 1).

### Secondary outcome measures for children

#### *Perceived social support*

Children’s perceived social support will be measured with adapted versions of the Social Support Inventory for adults (Called *Sociale Steun Lijst* (SSL) in Dutch) [37]. Children aged 12–16 years will complete the SSL youngster version [38]. Children aged 8–11 years will complete the Perceived Social Support Questionnaire 8–11 (PSSQ8-11) [39]. Both questionnaires will ask the children to express their perceived social support on three subscales: daily emotional support, support in problem situations and negative interactions. The questionnaires use a 4-point scale and consist of 19 items. Internal consistency is sufficient (Table 1). Children aged 5–7 years will complete the negative interactions subscale, using a 2-point scale (yes/no). This subscale consists of 7 items. This is the only reliable subscale for this age group (unpublished internal report by Snoeren & Hoefnagels), and no other age-appropriate questionnaire with sufficient psychometric properties is available for this age group.

#### *Perceived stress*

Children’s perceived stress will be measured with adapted versions of the Maastricht University Stress Instrument for Children (MUSIC) [40], which will be completed by children aged 12–16 years. Children aged 8–11 years will complete the Perceived Stress Questionnaire 8–11 (PSQ8-11) [39]. Both questionnaires ask children to express their perceived stress on two subscales: psychological stress and physiological stress. The questionnaires use a 4-point scale and consist of 20 items. Internal consistency is sufficient (Table 1). Children aged 5–7 years will complete an adapted version of the PSQ8-11, in which answering options are reduced to 'yes/no’ instead of the 4-point scale.

#### *Coping*

Children’s coping skills will be measured with two different questionnaires for children aged 8–11 and 12–16 years. No age-appropriate questionnaire with sufficient psychometric properties was available for the 5–7 age group. Children aged 12–16 years will complete the Coping Inventory for Stressful Situations (CISS) [41]. Children will be asked to rate the extent to which they use various coping skills in stressful situations, on three subscales: problem-focused coping, emotion-focused coping and avoidance. The CISS uses a 5-point scale and consists of 48 items. Internal consistency is good (Table 1). Children aged 8–11 years will complete the Dutch translation of the Children’s Coping Strategies Checklist [42] (Called *Coping Strategieën Lijst voor Kinderen* (CSLK) in Dutch [43]). Children will be asked to rate the extent to which they use various coping skills in stressful situations, on four subscales: active coping, distraction, avoidance and support seeking. The CSLK uses a 4-point scale and consists of 54 items. Internal consistency is good (Table 1).

#### *Self-esteem*

Children’s self-esteem will be measured with two adapted versions (a child version and an adolescent version) of the Self-Perception Profile for Adolescents [44] (referred to in Dutch as the *Competentie Beleveningsschaal voor Adolescenten* (CBSA) for children aged 12–16 years and the *Competentie Belevingsschaal voor kinderen* (CBSK) for children aged 8–11 years [45,46]). No age-appropriate questionnaire with sufficient psychometric properties was available for the 5–7 age group. Both questionnaires ask children to express their perceptions of six competencies: academic competence, social acceptance, athletic competence, physical appearance, behavioral conduct and global self-worth. The questionnaires use a 4-point scale and consist of 35 items. Internal consistency is good (Table 1).

In addition to the outcome measures described above, children will be asked about their disclosure of child maltreatment (did they disclose maltreatment to anyone, and if so to whom?) in a short interview after the questionnaire is completed.

### Secondary outcome measures for parents

#### *Quality of life*

Quality of life of the parents will be measured with the Dutch translation of the RAND 36-item Health Survey (RAND-36) [47], a short version of the RAND Health Insurance Study Questionnaire [48]. Parents will be asked to express their concerns on nine dimensions: physical functioning, social functioning, constraint due to physical problems, constraint due to emotional problems, mental health, energy, pain and perception of health. The questionnaire uses different multiple choice answering options for each item and consists of 11 items. Internal consistency is good (Table 2).

**Table 2:** Internal consistency of the Dutch translations of the questionnaires used to measure adult determinants

**Outcomes measure**	**Questionnaire**	**Cronbach’s alpha**	**Reference**
Quality of life	Rand-36	.71 – .92	Van der Zee & Sanderman, [47]
Perceived social support	SSL-Interactions	.90 – .93	Van Sonderen, [37]
	SSL-Discrepancies	.83 – .95	
	SSL-Negative interactions	.69 – .81	
Parental stress	NOSI-K	.97	Brock et al. [50]
Gender specific stress	FGRS	.93	Van Well et al. [51]
	MGRS	.90	
Coping	CISS	.82 – .87	Evers et al. [60]
Neighborhood perception	Neighborhood Characteristics Scale	NA	NA
Life events	VMG	.80	Veerman et al. [52]

#### *Parental stress*

Parental stress will be measured with an adapted version and translation of the Parenting Stress Index [49] (referred to in Dutch as *Nijmeegse Ouderlijke Stress Index*, short version (NOSI-K) [50]. Parents will be asked to express their agreement with several hypotheses concerning the upbringing of their child. The NOSI-K uses a 6-point scale and consists of 25 items. Internal consistency is good (Table 2).

#### *Gender-specific stress*

Gender-specific stress will be measured with the Dutch translation of the Feminine Gender Role Stress (FGRS) for women and the Masculine Gender Role Stress (MGRS) for men [51]. Parents will be asked to express the extent to which they perceive several situations as stressful. The FGRS consists of subscales for non-emotional relationships, bodily unattractiveness, victimization, assertive behavior and non-caring behavior. The MGRS consists of subscales for bodily inadequateness, emotional expressionlessness; being subordinate to women, intellectual inferiority and not being able to perform. Both questionnaires use a 6-point scale and consist of 40 items. Internal consistency is good (Table 2).

#### *Perceived social support*

Perceived social support will be measured with the Social Support Inventory for adults (Called *Sociale Steun Lijst* (SSL) in Dutch) [37]. Parents will be asked to express their perceived social support on three dimensions: SSL-Interactions (SSL-I), SSL-Discrepancies (SSL-D) and SSL-Negative interactions (SSL-N) The dimensions SSL-I and SSL-D both consist of seven subscales (daily emotional support, emotional support in problem situations, appreciation, instrumental interactions, social companionship, informative support) and has 34 items. The SSL-N has 7 items. All dimensions use a 4-point scale. Internal consistency is good (Table 2).

#### *Coping*

Coping will be measured with the Coping Inventory for Stressful Situations (CISS) [41]. Parents will be asked to rate the extent to which they use several coping skills in stressful situations on three subscales: problem-focused coping, emotion-focused coping and avoidance. The CISS uses a 5-point scale and consists of 48 items. Internal consistency is good (Table 2).

#### *Life events*

Life events will be recorded with the Dutch *Vragenlijst Meegemaakte Gebeurtenissen* (VMG) [52]. Parents will be asked to report if, how many times and when for the last time, various positive or negative life events have occurred in the child’s life. In addition, if a life event has occurred, the parent will be asked to rate the child’s perception of it as positive or negative. The VMG consist of 24 items. Internal consistency is good (Table 2).

#### *Neighborhood perception*

Neighborhood perception will be measured with the Dutch translation of the Neighborhood Characteristics Scale [53]. Parents will be asked to express their agreement with several hypotheses concerning their neighborhood on two subscales: physical neighborhood disorder and social neighborhood disorder. The Neighborhood Characteristics Scale uses a 5-point scale and consists of 18 items. The original scale has good psychometric properties (α .92). Information on the psychometric properties of the Dutch translation is not available.

In addition to the above outcome measures, parents will be asked to complete a recording form to collect information on gender, age, ethnic background, parental education level, current living situation and employment status, net income, financial problems, alcohol use and health care information.

### Analyses

First a non-response analysis, using univariate analysis, will be carried out to compare characteristics of the included participants with the characteristics of the entire population of children aged 5–16 years reported to the seven participating AMKs within the recruitment period of this study. The following characteristics will be studied: age of the child, gender of the child, ethnicity, living situation, type of maltreatment as verified by the AMK, outcome of the AMK procedure.

Missing data at follow-up will be imputed using regression imputation. Prior to the analyses the possible influence of (1) the different AMKs (as families will be derived from 7 AMKs), and (2) the time between the report to the AMK and the completion of the questionnaires will be examined.

The first research question, regarding the mental health and quality of life of maltreated children over a 1.5 year period, will be addressed using a longitudinal multi-level analyses (measurements nested within respondents, i.e. children and parents/caregivers, respectively) to assess changes in mental health and quality of life over time [54]. The second research question, regarding the influence of relevant determinants on the mental health and quality of life of maltreated children, will be addressed by exploring the potential effects of these determinants on the outcome variables by testing the interaction between potential effect modifiers at T0 and the outcome variables at Tf-upx (where 'f-upx’ indicates the successive moments of measurement during follow–up).

To answer the third research question, regarding differences in mental health and quality of life outcomes between children from families of Dutch origin with children from families originating from Morocco and Suriname, 'ethnicity’ will be entered as a covariate in the analysis.

### Collaboration

This study is a joint project of the Trimbos institute, the Netherlands Institute for Mental Health and Addiction (Utrecht) and the CAHPRI School for Public Health and Primary Care, Maastricht University and VU University Amsterdam. The research is funded by ZonMw (project 15700.2012) and is registered in the Netherlands Trial Register, part of the Dutch Cochrane Centre (NTR3674). The Dutch Medical Ethics Committee for Mental Health Care (METiGG) has approved the study (NL31267.097.10).

## Discussion

Although AMKs play and important role in the Netherlands in the attempt to end child maltreatment and to provide the necessary care for maltreated children and their families, the children’s mental health and quality of life after AMK involvement has not yet been studied. This gap in the available knowledge, as well as prior research showing the negative impact of child maltreatment on the mental health and quality of life of children, means there is a need for the present study. The results of this study will primarily provide information on changes in the mental health and quality of life of maltreated children over a period of 1.5 years after an AMK becomes involved. Secondly, the study will explore determinants that positively or negatively influence the mental health and/or quality of life of the maltreated children. These results will help to identify the children that are most vulnerable to developing mental health problems and will define determinants that can be influenced to improve these children’s mental health and quality of life or to prevent additional harm. Thirdly, the results will provide information on the differences in mental health and quality of life outcomes between families of Dutch origin and families originating from Morocco and Suriname.

This study has several strengths. First, this study will be performed among maltreated children and their parents shortly after they have been reported to an AMK, which may reduce recall bias. Since there is a lack of studies examining outcomes shortly after or during the actual maltreatment and outcomes may be expected to be different during or shortly after abuse in comparison with years later, this study can contribute to the knowledge about the short-term effects of maltreatment.

Another strength is the use of child self-reports. Many studies have used proxy informants when collecting data on children’s mental health and quality of life. However, the use of proxies is not always the best option when measuring subjective constructs, and research findings have shown discrepancies between child and parent reports [26-29]. For example, quality of life studies have reported better agreement between child and proxy reports on the physical, more observable, dimensions and poor agreement on the social and emotional, non-observable, quality of life dimensions [26]. As a consequence, the value of obtaining children’s self-reports is increasingly recognized and several studies have examined the accuracy and reliability of child reports. These studies have shown that children can provide reliable and accurate information as informants [26,55,56]. The present study will examine the outcomes of maltreated children aged 5–16 years, using child self-reports as much as possible.

Quality of life has become an important topic of research on the adverse effects of childhood maltreatment, as the concept of quality of life adds a subjective component to objective, clinical measures [11-14]. Self-reported quality of life will be examined as one of the main objectives of this study. This should be useful, as quality of life research focusing on children, especially maltreated children, has so far been scarce [15-17].

This study also has several limitations. First, selection bias cannot be ruled out. Participation is voluntary and families will be recruited shortly after a report of suspected child maltreatment has been filed with an AMK. This can be expected to be a stressful time for most families. In addition, families might be inclined to refuse participation in view of the perceived accusation and/or stigmatization by the child maltreatment report [57]. This may make it harder to find participants for this study, which may lead to a risk of selection bias. To increase the likelihood of success, the researchers will remain in close contact with the participating AMKs during the recruitment process, so they can adjust the process if necessary. In addition, a non-response analysis will be performed to examine the presence of this possible selection bias. Secondly, there is a risk of considerable loss to follow-up. One reason may be that families of maltreated children tend to move more frequently than non-maltreated children and their families [58]. Families may forget to forward their new address and phone number. To minimize loss to follow-up due to changes in contact information, participants will be asked to provide various kinds of contact information. Another possible reason is that many families reported to AMKs are 'multi-problem’ families [59], so there might be new and/or additional problems at follow-up which might cause families to drop out of the study.

To conclude, this study will be the first to provide information on mental health and quality of life outcomes of maltreated children over a 1.5 year period after being reported to an AMK. In addition, it will explore determinants that influence mental health and quality of life outcomes, and will provide information on the influence of ethnicity on maltreatment outcomes. Results will contribute to the knowledge that is required to provide high-quality care to maltreated children.

## Competing interests

All authors declare that they have no competing interests.

## Authors’ contributions

All authors participated in describing the design of this study. CH, SMAAE and FLW obtained funding for this study. All authors have read and corrected draft versions of the manuscript and approved the final manuscript.

## Pre-publication history

The pre-publication history for this paper can be accessed here:

http://www.biomedcentral.com/1471-2458/13/942/prepub
